# Observation and control of Casimir effects in a sphere-plate-sphere system

**DOI:** 10.1038/s41467-022-33915-4

**Published:** 2022-10-18

**Authors:** Zhujing Xu, Peng Ju, Xingyu Gao, Kunhong Shen, Zubin Jacob, Tongcang Li

**Affiliations:** 1 Department of Physics and Astronomy, Purdue University, West Lafayette, IN 47907 USA; 2 Elmore Family School of Electrical and Computer Engineering, Purdue University, West Lafayette, IN 47907 USA; 3 Birck Nanotechnology Center, Purdue University, West Lafayette, IN 47907 USA; 4 Purdue Quantum Science and Engineering Institute, Purdue University, West Lafayette, IN 47907 USA

**Keywords:** Quantum optics, Single photons and quantum effects, Quantum optics, NEMS

## Abstract

A remarkable prediction of quantum field theory is that there are quantum electromagnetic fluctuations (virtual photons) everywhere, which leads to the intriguing Casimir effect. While the Casimir force between two objects has been studied extensively for several decades, the Casimir force between three objects has not been measured yet. Here, we report the experimental demonstration of an object under the Casimir force exerted by two other objects simultaneously. Our Casimir system consists of a micrometer-thick cantilever placed in between two microspheres, forming a unique sphere-plate-sphere geometry. We also propose and demonstrate a three-terminal switchable architecture exploiting opto-mechanical Casimir interactions that can lay the foundations of a Casimir transistor. Beyond the paradigm of Casimir forces between two objects in different geometries, our Casimir transistor represents an important development for controlling three-body virtual photon interactions and will have potential applications in sensing and information processing.

## Abstract (web summary)

Experimental studies of the Casimir effect have involved only interactions between two bodies so far. Here, the authors observe a micrometer-thick cantilever under the Casimir force exerted by microspheres from two sides simultaneously.

## Introduction

The interaction between three objects give rise to many fascinating phenomena such as chaos of astronomical objects^1^, Efimov bound states of ultracold atoms^2^, and frustrated states of quantum spin systems^3^. It is intriguing to consider the potential of three-body interactions arising solely from quantum electromagnetic fluctuations (virtual photons)^4–6^. As quantum electromagnetic fluctuations exist everywhere in the universe and in human-made devices, the Casimir effect is ubiquitous. However, the Casimir effect is often neglected or treated as a detrimental effect in micro-electromechanical systems and nano-electromechanical systems^7^. It will be beneficial to develop useful applications of the Casimir effect. One application of the Casimir effect is to provide a new approach to couple mechanical resonators^8^. Different from optomechanical coupling with real photons in cavity optomechanics^9–12^, optomechanical coupling with virtual photons will not suffer from cavity loss and thus will not require a high-quality cavity. Compared to electrostatic coupling, the Casimir effect does not require charge and will work for both conductors and insulators. Compared to the optical force, the Casimir force works for both transparent and opaque materials. Meanwhile, the use of the Casimir force does not prevent the use of the electrostatic force and the optical force. They may be combined to achieve more advanced functions. In addition, the Casimir effect is energy efficient as it does not require a voltage source or a light source to maintain, which will be particularly important for devices operating in the quantum regime at low temperatures.

Recently, the Casimir effect was used to increase the quality factor of a mechanical resonator^13^ and couple two separate mechanical resonators^14,15^. In addition, the Casimir effect has been utilized to realize nonlinear oscillation^16^, quantum trapping and self-assembling^17,18^. While the paradigm of Casimir effect between two objects has been extensively explored^19–26^, the Casimir force between three macroscopic objects has not been detected yet. The Casimir effect between three objects will be important in studying gravity at short distances as a metal film is often used to separate two test masses to shield electrostatic interactions^27–29^. Precision measurement of the Casimir effect has been used to search for a new force beyond the standard model^30^. Beyond its fundamental interest, a Casimir system with three objects can open the route to realize crucial technological building blocks such as a transistor-like three-terminal device with quantum vacuum fluctuations.

**Fig. 1:**
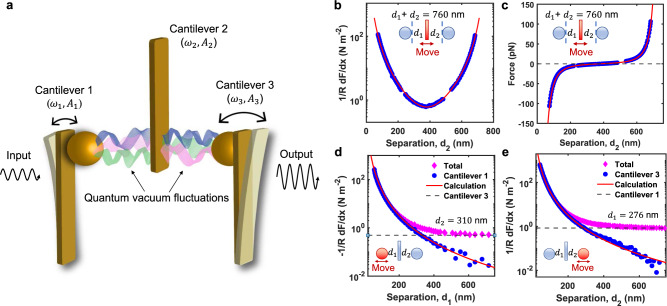
Casimir interaction between three optomechanical resonators. **a** Three modified cantilevers with resonant frequencies *ω*_1_, *ω*_2_ and *ω*_3_ experience Casimir force between each two nearby surfaces. The vibration amplitudes of three cantilevers are denoted as *A*_1_, *A*_2_ and *A*_3_. Additional parametric modulations are applied on the center cantilever to couple them by the Casimir effect. We can switch on and off the Casimir coupling between cantilever 1 and cantilever 3 by controlling the parametric modulations. In addition, we can amplify the energy transfer through Casimir effect by adding an extra gain to cantilever 2. **b** Measured Casimir force gradient on cantilever 2 (center) as a function of its position when the other two surfaces are fixed such that *d*_1_ + *d*_2_ = 760 nm. The blue circles are experimental measurements and the red solid line are the theoretical prediction. **c** The measured Casimir force on cantilever 2 is shown as a function of *d*_2_. **d** Measured Casimir force gradient experienced by cantilever 2 as a function of *d*_1_ when *d*_2_ is fixed at 310 nm. The red diamonds are the total force gradient $-\frac{1}{R}\frac{dF}{dx}$ measured from cantilever 2. The blue circles are the force gradient contributed from cantilever 1. The red solid curve is the theoretical prediction of the interaction between cantilever 1 and 2. The gray dashed line is the theoretical prediction of the interaction between cantilever 2 and 3 and hence it is independent of *d*_1_ under additivity approximation. **e** Measured Casimir force gradient on cantilever 2 as a function of *d*_2_ when *d*_1_ is fixed at 276 nm.

In this article, we report the experimental demonstration of an object under the Casimir force exerted by two other objects simultaneously. We also propose and demonstrate a three-terminal Casimir system that can switch and amplify quantum-vacuum-mediated energy transfer, in analogy to a field effect transistor. Our unique sphere-plate-sphere Casimir system consists of three closely-spaced optomechanical oscillators, as shown in Fig. 1a. A micrometer-thick cantilever is placed in between two microspheres which are attached to two other cantilevers. Their motions are monitored by three independent fiber-optic interferometers. There are random quantum vacuum fluctuations between them and hence each cantilever experiences a separation-dependent Casimir force. We first measure the Casimir force in this sphere-plate-sphere system. We then apply parametric modulation on cantilever 2 to couple their motion by the Casimir effect. In this way, energy can flow from cantilever 1 to cantilever 2 and to cantilever 3. The center cantilever serves as a gate for controlling the energy transfer through the Casimir effect. By adding gain to the center cantilever with active feedback, we also realize amplification of the quantum-fluctuation-mediated energy transfer. Our Casimir transistor will have promising application in sensing^31,32^ and information processing^33,34^.

## Results

### Casimir force in the sphere-plate-sphere configuration

We first measure the Casimir force in our sphere-plate-sphere system (Fig. 1). Assuming three surfaces are all made of ideal conductive metal and they are sufficiently thick, the Casimir force on the center one is^23^:

1 $${F}_{2,C}^{0}=\frac{{\pi }^{3}\hslash c}{360}\left(\frac{{R}_{1}}{{d}_{1}^{3}}-\frac{{R}_{2}}{{d}_{2}^{3}}\right),$$

where *d*_1_ and *d*_2_ are the separations between cantilever 1 and cantilever 2, and the separation between cantilever 2 and cantilever 3 as shown in the inset of Fig. 1b. *R*_1_ and *R*_2_ are the radii of the sphere on cantilever 1 and cantilever 3, respectively. The Casimir force strongly depends on the separations and is highly nonlinear. Such a large nonlinearity is crucial for achieving parametric coupling as described in the next section. The Casimir interaction between real materials can be calculated by the Lifshitz theory^15,35^. Our custom Matlab codes to calculate the Casimir interaction in a sphere-plate-sphere system are provided in the Supplementary Software. We use the dynamic force measurement scheme to measure the Casimir force. More details about the calculation of Casimir interaction in our system can be found in Methods and Supplementary Note 1. The detailed force measurement schemes and results are presented in Supplementary Note 2 and Supplementary Figs. 2–5.

The measured Casimir force gradient on cantilever 2 in our three-body system is shown in Fig. 1b. We fix the position of cantilever 1 and 3 such that *d*_1_ + *d*_2_ = 760 nm. Meanwhile, we change the position of the cantilever 2 (center). As the center cantilever moves from left side to the right side, the gradient meets the lowest value when *d*_1_ = *d*_2_ if *R*_1_ = *R*_2_. At this specific separation, the net Casimir force on cantilever 2 is zero. The calculation based on Lifshitz’s formula and proximity force approximation is shown in the solid red curve. The measurement is in good agreement with the calculation. We also show the measured Casimir force gradient on cantilever 2 when separation *d*_1_ is changed by moving cantilever 1 in Fig. 1d, and similarly when separation *d*_2_ is changed by moving cantilever 3 in Fig. 1e. While there have been many studies of Casimir interaction between two objects, our work reports the measurement of the Casimir force between three separate objects. It opens up the possibility for studying Casimir interaction between more complicated configurations, and can study the nonadditivity nature^36–39^ of the Casimir interaction by reducing the thickness of the center plate (see Supplementary Fig. 1).

### Casimir vibration coupling

**Fig. 2:**
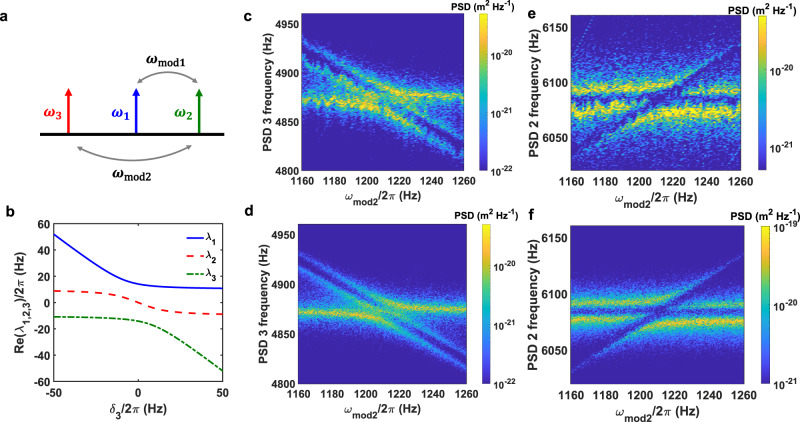
Coupling the vibrations of three cantilevers with the Casimir effect. **a** Parametric modulation of the Casimir interaction is applied in our system. When *ω*_mod1_ = *ω*_2_ − *ω*_1_, cantilever 1 and cantilever 2 are coupled. Similarly, cantilever 2 and cantilever 3 are coupled when *ω*_mod2_ = *ω*_2_ − *ω*_3_. Here ${\omega }_{{{{{{{{\rm{mod}}}}}}}}1,2}$ is the modulation frequency. **b** Three eigenvalues of the Hamiltonian in Eq. (2) as a function of *δ*_3_ when *δ*_2_ = 0 and ∣*g*_12_∣ = ∣*g*_23_∣ = 2*π* × 20 Hz. Here *δ*_2_ = *ω*_1_ + *ω*_mod1_ − *ω*_2_ and *δ*_3_ = *ω*_1_ + *ω*_mod1_ − *ω*_mod2_ − *ω*_3_ are the detuning of the system. *g*_12_ and *g*_23_ are the coupling strengths between cantilever 1 and cantilever 2, and between cantilever 2 and cantilever 3, respectively. **c** Measured power spectrum density (PSD) of cantilever 3 as a function of the modulation frequency *ω*_mod2_. **e** PSD of cantilever 2 as a function of *ω*_mod2_. The modulation amplitudes are *δ*_d1_ = 10.4 nm and *δ*_d2_ = 14.1 nm. The modulation frequency *ω*_mod1_ is fixed at 440 Hz. **d**, **f** The simulated PSD for two cantilevers. The separations are *d*_10_ = 88 nm and *d*_20_ = 90 nm.

We now use the Casimir effect to efficiently couple the motions of three cantilevers for realizing a more advanced Casimir-based device. The natural frequencies and damping rates of three cantilevers are *ω*_1_ = 2*π* × 5661 Hz, *ω*_2_ = 2*π* × 6172 Hz, *ω*_3_ = 2*π* × 4892 Hz, *γ*_1_ = 2*π* × 3.22 Hz, *γ*_2_ = 2*π* × 6.06 Hz, and *γ*_3_ = 2*π* × 3.58 Hz when they are far apart. These frequencies shift under Casimir interaction. The direct Casimir coupling strength between three cantilevers is smaller than the frequency differences between them. To solve this issue, we use parametric coupling^15,40^ by modulating the separation between each two cantilevers at a slow rate *ω*_mod1,2_ and a modulation amplitude *δ*_d1,2_. This is achieved by changing the position of the cantilever 2 as ${\delta }_{{{{{{{{\rm{d1}}}}}}}}}\cos ({\omega }_{{{{{{{{\rm{mod1}}}}}}}}}t)+{\delta }_{{{{{{{{\rm{d2}}}}}}}}}\cos ({\omega }_{{{{{{{{\rm{mod2}}}}}}}}}t)$. Such parametric modulation effectively couples three cantilevers when *ω*_mod1_ = ∣*ω*_1_ − *ω*_2_∣ and *ω*_mod2_ = ∣*ω*_3_ − *ω*_2_∣, as shown in Fig. 2a. Different from direct coupling that requires identical resonant frequencies, parametric coupling provides more freedom to couple different resonators. Under the parametric coupling scheme, the simplified Hamiltonian of the three-cantilever system in the interaction picture is (see Methods and Supplementary Note 4 for detailed derivations)^15^:

2 $$H=\left(\begin{array}{lll}-i\frac{{\gamma }_{1}}{2}&\frac{{g}_{12}}{2}&0\\ \frac{{g}_{12}}{2}&-i\frac{{\gamma }_{2}}{2}-{\delta }_{2}&\frac{{g}_{23}}{2}\\ 0&\frac{{g}_{23}}{2}&-i\frac{{\gamma }_{3}}{2}-{\delta }_{3}\end{array}\right).$$

where *γ*_1,2,3_ denote the damping rates of the three cantilevers. ${g}_{12}=\frac{{{{\Lambda }}}_{1}}{2\sqrt{{m}_{1}{m}_{2}{\omega }_{1}{\omega }_{2}}}$ and ${g}_{23}=\frac{{{{\Lambda }}}_{2}}{2\sqrt{{m}_{2}{m}_{3}{\omega }_{2}{\omega }_{3}}}$ are the coupling strengths between cantilever 1 and cantilever 2, and between cantilever 2 and cantilever 3, respectively. Here we have ${{{\Lambda }}}_{1}=\frac{{d}^{2}{F}_{C}}{d{x}^{2}}{|}_{{d}_{10}}{\delta }_{{{{{{{{\rm{d1}}}}}}}}}$ and ${{{\Lambda }}}_{2}=\frac{{d}^{2}{F}_{C}}{d{x}^{2}}{|}_{{d}_{20}}{\delta }_{{{{{{{{\rm{d2}}}}}}}}}$. Here *d*_10,20_ is the equilibrium separation when there is no modulation applied. *δ*_2_ = *ω*_1_ + *ω*_mod1_ − *ω*_2_ and *δ*_3_ = *ω*_1_ + *ω*_mod1_ − *ω*_mod2_ − *ω*_3_ are the detuning of the system which depend on the modulation frequencies. The eigenvalues of this Hamiltonian near resonant coupling conditions are shown in Fig. 2b. We can observe a clear two-fold anti-crossing when the detunings *δ*_3_ = *δ*_2_ = 0.

Our experimental results of the level repulsion behavior due to the Casimir coupling between three cantilevers are show in Fig. 2c, e. We study this behavior experimentally by scanning the power spectrum densities (PSD) of cantilever 3 (Fig. 2c) and cantilever 2 (Fig. 2e) as a function of the modulation frequency *ω*_mod2_ when *ω*_mod1_ = ∣*ω*_1_ − *ω*_2_∣. Figure 2e shows three branches which correspond to the hybrid modes of three cantilevers after being projected to cantilever 2. Since *ω*_mod1_ is fixed at the resonant value that can couple cantilever 1 and cantilever 2, we notice a clear anti-crossing behavior (a horizontal dark line around 6080 Hz) independent of *ω*_mod2_. The two horizontal branches describe the coupled motion of cantilevers 1 and 2. When we vary *ω*_mod2_, we also observe an inclined branch with a frequency *ω*_3_ + *ω*_mod2_ which corresponds to the motion of cantilever 3. When this inclined branch intersects with the other two branches at *ω*_mod2_ = ∣*ω*_3_ − *ω*_2_∣, a more complicated level repulsion is observed. One mode disappears in the PSD of cantilever 2 as this mode only involves the motion of cantilever 1 and 3. More detailed discussions about the eigenvalues and PSD of the system is included in Supplementary Note 4. Numerical simulation results are shown in Fig. 2d, f, which agree well with experimental results. Thus we have strongly coupled the motions of three objects with quantum vacuum fluctuations.

### Casimir switch

**Fig. 3:**
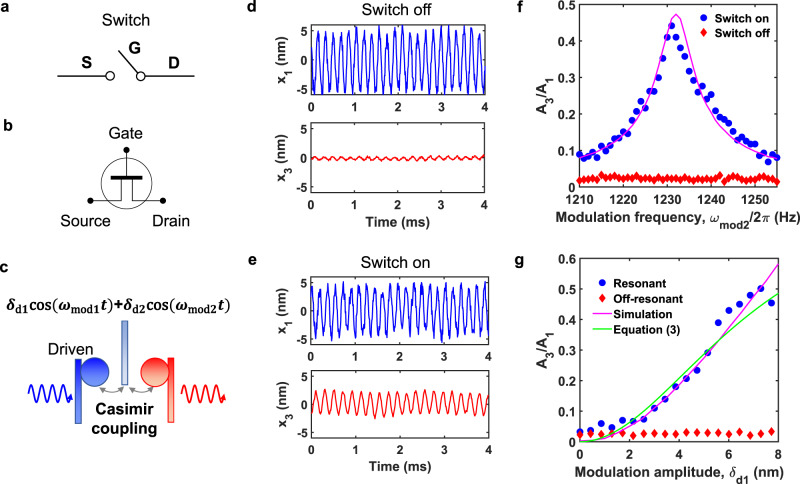
Switching quantum-fluctuation-mediated energy transfer. **a** A symbolic switch. **b** A symbolic field effect transistor. **c** The quantum-fluctuation-mediated energy transfer between cantilever 1 and 3 can be switched on and off by the modulation on cantilever 2. The position of cantilever 2 is modulated with modulation amplitudes *δ*_d1,2_ and modulation frequencies *ω*_mod1,2_. **d** Measured displacement of two cantilevers (*x*_1_ and *x*_3_) when modulation is off. **e** Measured displacement of two cantilevers when modulation is on. Energy from cantilever 1 is transferred efficiently to cantilever 3. Here *ω*_mod1,2_ = 2*π* × 465 Hz, *ω*_mod2_ = 2*π* × 1230 Hz, *δ*_d1_ = 6.0 nm, and *δ*_d2_ = 8.5 nm. The separations are *d*_10_ = 100 nm and *d*_20_ = 105 nm. **f** The transduction ratio *A*_3_/*A*_1_ is shown as a function of the modulation frequency *ω*_mod2_ when *ω*_mod1_ is on resonant. *A*_1_ and *A*_3_ are the vibrational amplitudes. The blue circles and the red diamonds correspond to the switch on and off case, respectively. The magenta solid line is the simulation. **g** The transduction ratio *A*_3_/*A*_1_ as a function of modulation amplitude *δ*_d1_ when *ω*_mod2_ = 2*π* × 1231 Hz (on resonant, blue circles) and *ω*_mod2_ = 2*π* × 1150 Hz (off resonant, red diamonds). *ω*_mod1_ is on resonant for both cases. The magenta solid curve is the simulation and the green solid curve is the calculation based on Eq. (3).

**Fig. 4:**
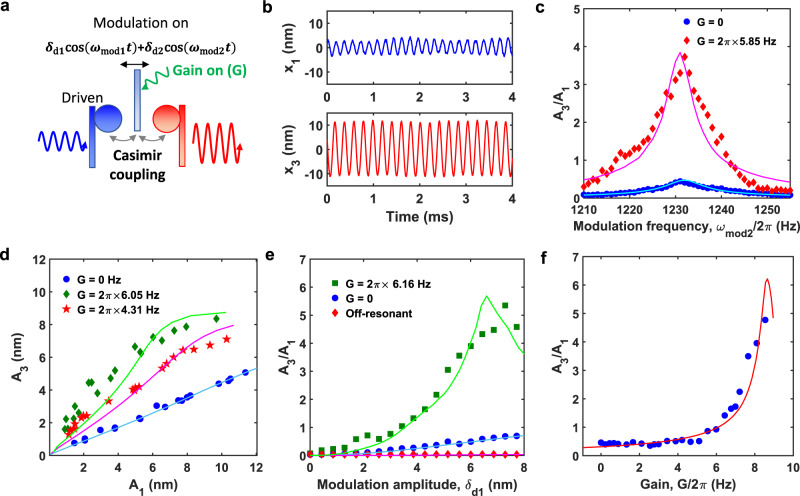
Amplifying quantum-fluctuation-mediated energy transfer. **a** An extra gain *G* is applied on cantilever 2 by feedback control together with parametric modulation. **b** The signal on cantilever 1 (*x*_1_) is transmitted to cantilever 3 (*x*_3_) with amplification. **c** The transduction ratio *A*_3_/*A*_1_ is shown as a function of modulation frequency *ω*_mod2_ when the gain is on (red diamonds) and off (blue circles). *ω*_mod1_ is on resonant for both cases. *G* is the gain coefficient on cantilever 2. Under the extra gain, the damping rate of cantilever 2 becomes *γ*_2_ = *γ*_20_ − *G*, where *γ*_20_ is the natural damping rate. The cyan and magenta solid curve are the simulations. **d** The vibrational amplitude *A*_3_ is shown as a function of amplitude *A*_1_ for three different gain coefficients *G*. The parametric modulation is applied resonantly at the same time. The green, magenta and blue solid line are the simulated results for three different gain coefficients. **e** The ratio *A*_3_/*A*_1_ is shown as a function of modulation amplitude *δ*_d1_ for cases with gain, no gain, and off-resonant modulation. *δ*_d2_ = 1.42*δ*_d1_. **f** The transduction ratio *A*_3_/*A*_1_ (blue circles) is shown as a function of the extra feedback gain *G* applied on cantilever 2. The red solid curve is the simulation.

Our three-terminal Casimir system enables switching (Fig. 3a) and amplifying quantum-fluctuation-mediated energy transfer in analogy to a field effect transistor (Fig. 3b). The quantum-fluctuation-mediated energy transfer between cantilever 1 and 3 can be easily switched on and off by controlling the modulation on cantilever 2 (Fig. 3c). When *ω*_mod1_ and *ω*_mod2_ are on resonance, vibration energy from cantilever 1 can be transferred to cantilever 3 efficiently (Fig. 3e). However, when the modulation is off, the excitation on cantilever 1 can not be transferred to cantilever 3 efficiently (Fig. 3d). In Fig. 3f, the measured amplitude ratio *A*_3_/*A*_1_ is shown as a function of modulation frequency *ω*_mod2_ for both switch on and off cases. We notice that the amplitude ratio can achieve up to 0.44 when the modulation is on resonance, and close to zero when the modulation if off resonance. Thus we can switch on and off the quantum-fluctuation-mediated energy transfer with high contrast. The separations in Figs. 3 and 4 are larger than those separations in Fig. 2 and give smaller coupling strengths: *g*_12_ = 2*π* × 3.5 Hz and *g*_23_ = 2*π* × 3.9 Hz. These coupling strengths are comparable to the damping rates of the cantilevers so there is no mode splitting in this case.

Under the steady state when the cantilever 1 is driven at *ω*_1_ with a small amplitude and the parametric modulation on cantilever 2 is on resonant, the transduction ratio *A*_3_/*A*_1_ in this three-cantilever Casimir system is (see Methods):

3 $$\frac{{A}_{3}}{{A}_{1}}=\left|\frac{{{{\Lambda }}}_{1}{{{\Lambda }}}_{2}}{4{m}_{2}{m}_{3}{\omega }_{2}{\omega }_{3}{\gamma }_{2}{\gamma }_{3}+{{{\Lambda }}}_{2}^{2}} \right|.$$

where ${{{\Lambda }}}_{1}=\frac{{d}^{2}{F}_{C}}{d{x}^{2}}{|}_{{d}_{10}}{\delta }_{{{{{{{{\rm{d1}}}}}}}}}$ and ${{{\Lambda }}}_{2}=\frac{{d}^{2}{F}_{C}}{d{x}^{2}}{|}_{{d}_{20}}{\delta }_{{{{{{{{\rm{d2}}}}}}}}}$. For Fig. 3, the cantilever 1 is driven at 5649 Hz, which is its resonant frequency in this situation. In Fig. 3g, the measured transduction ratio *A*_3_/*A*_1_ is shown as a function of modulation amplitude *δ*_d1_ when *δ*_d2_ = 1.42*δ*_d1_. The transduction ratio is close to zero when the parametric modulation is off-resonant. As expected, the ratio *A*_3_/*A*_1_ increases when *δ*_d1_ increases under resonant coupling. Our experimental results agree well with Eq. (3) and numerical simulation results (Fig. 3g).

### Casimir amplification

To realize a Casimir transistor with high efficiency, we introduce an extra gain to the system (Fig. 4a) to amplify the quantum-fluctuation-mediated energy transfer. The extra gain is applied to cantilever 2 by feedback control such that the damping rate of cantilever 2 becomes *γ*_2_ = *γ*_20_ − *G*, where *γ*_20_ is the natural damping rate of cantilever 2 and *G* is the gain coefficient (More details can be found in Supplementary Note 3 and Supplementary Fig. 6). *γ*_2_ becomes negative when *G* > *γ*_20_. Based on Eq. (3), the transduction ratio *A*_3_/*A*_1_ increases when *γ*_2_ decreases. Under such condition, energy from cantilever 1 is first transferred to cantilever 2 and get amplified and then transferred to cantilever 3. For example, we apply a fixed gain to cantilever 2 such that *G* = 2*π* × 8.73 Hz to realize the amplification of energy transfer, as shown in Fig. 4b. Other parameters are the same as those in Fig. 3e.

Figure 4c shows amplification of quantum-fluctuation-mediated energy transfer with our Casimir transistor. When a gain is applied to cantilever 2, energy transfer from cantilever 1 to cantilever 3 shows a similar resonant behavior as the no-gain case, but has an improvement by a factor of 8 on the transduction ratio . The additional gain improves the quantum-fluctuation-mediated energy transfer efficiency significantly. As expected, the transduction ratio *A*_3_/*A*_1_ increases when the parametric modulation amplitude (Fig. 4e) or the gain coefficient (Fig. 4d, f) increases until the system becomes unstable when the modulation amplitude or the gain is too large. Thus we have demonstrated amplification in a three-terminal Casimir system. The amplification function will be crucial for future applications of Casimir-based devices. For example, Casimir parametric amplification has been theoretically proposed for zeptometer metrology^31^ and ultrasensitive magnetic gradiometry^32^.

## Discussion

We have measured the Casimir interaction between three objects, and demonstrated efficient coupling of three optomechanical resonators with virtual photons. Compared to the conventional optomechanical coupling with real photons in a high-Q cavity^9,10^, optomechanical coupling with virtual photons^8,41^ does not need a high-Q cavity. Inspired by a field effect transistor, we also demonstrate switching and amplifying quantum-fluctuation-mediated energy transfer in our three-terminal Casimir system. As proposed by former theoretical studies, Casimir-based amplification and switching will have applications in sensing^31,32^ and information processing^33,34^. The realization of Casimir coupling between three resonators is an important step toward realizing a scalable Casimir array. The three resonators can also be modified to realize a circulator in the future, which can have non-reciprocity between any two of the three resonators^42^. By reducing the thickness of the center cantilever, the system can be used to study non-pairwise additive effects^38,39^. The Casimir effect between three objects is also important in studying gravity at short distances^27–29^.

## Methods

### Casimir force calculation

At a finite temperature, the Casimir interaction comes from both quantum and thermal fluctuations. At temperature T and separation *x*, the Casimir energy per unit area between two surfaces is given by^35^:

4 $$\begin{array}{lll}E(x,T)=\frac{{k}_{B}T}{2\pi }{\mathop{\sum }\limits_{l=0}^{\infty }}{}^\prime\int\nolimits_{0}^{\infty }{k}_{\perp }d{k}_{\perp }\{\ln [1-{r}_{TM}^{2}(i{\xi }_{l},{k}_{\perp }){e}^{-2xq}]+\ln [1-{r}_{TE}^{2}(i{\xi }_{l},{k}_{\perp }){e}^{-2xq}]\},\end{array}$$

where ${\xi }_{l}=\frac{2\pi {k}_{B}Tl}{\hslash }$ is the Matsubara frequency and ${k}_{\perp }=\sqrt{{k}_{x}^{2}+{k}_{y}^{2}}$ is the wave vector parallel to the surface. *r*_*TE*_(*iξ*_*l*_, *k*_⊥_) and *r*_*TM*_(*iξ*_*l*_, *k*_⊥_) are reflection coefficients of the transverse-electric and transverse-magnetic modes. The separation between two surfaces is far smaller than the dimensions of the cantilever and the sphere. Therefore, we can apply the proximity-force approximation and the Casimir force between a sphere with radius *R* and a plate is *F*_*C*_(*x*, *T*) = − 2*πRE*(*x*, *T*). The calculation in ref. 15 has shown that the contribution from thermal fluctuations at room temperature is less than 4% when the separation is less than 800 nm. Thus, the Casimir interaction in our system is dominated by quantum vacuum fluctuations. In our system, the thickness of the center cantilever is 1 μm and the typical separation in our measurement is from 50 to 800 nm. Under such condition, the contribution from the nonadditivity is negligible compared to the sum of the pair potential and hence we take the additivity approximation^36^. Under the thermal equilibrium, the force on the center cantilever can be simplified as *F*_2,*C*_ = − *F*_*C*_(*d*_1_, *T*) + *F*_*C*_(*d*_2_, *T*). Under the additivity approximation, the force gradient between cantilever 1 and cantilever 2 is calculated by subtracting the force gradient between cantilever 2 and cantilever 3 from the total gradient experienced by cantilever 2, as shown in Fig. 1d.

### Experimental setup and force measurement

In the experiment, we use three modified AFM cantilevers to build the three-terminal Casimir system. The left and right cantilever has a dimension of 450 × 50 × 2 μm^3^. The center cantilever has a dimension of 500 × 100 × 1 μm^3^. Two 70-μm-diameter polystyrene spheres are attached to the free end of the left and right cantilevers to create the sphere-plate-sphere geometry. Additional 100-nm-thick gold layers are coated on both the sphere and cantilever surfaces.

During the measurement, we use phase-lock loop to track the resonant frequency in the presence of the Casimir interaction. Then we can get the force gradient as $\frac{dF}{dx}=-2k\frac{\delta \omega }{\omega }$, where *k* is the spring constant of the cantilever, *δω* is the frequency shift in the presence of the interaction and *ω* is the natural resonant frequency. The separation between each two surfaces is calibrated by the electrostatic force. The frequency shift due to the electrostatic force and the Casimir force is ${{\Delta }}\omega=-\frac{\omega }{2k}\frac{\pi {\epsilon }_{0}R}{{x}^{2}}[{({V}_{{{{{{{{\rm{ext}}}}}}}}}-{V}_{{{{{{{{\rm{c}}}}}}}}})}^{2}+{V}_{{{{{{{{\rm{rms}}}}}}}}}^{2}]-\frac{\omega }{2k}\frac{d{F}_{C}}{dx}$, where *V*_ext_ is the external voltage applied on the surface, *V*_*c*_ is the patch potential, *V*_rms_ is the rms voltage fluctuations. $\frac{d{F}_{C}}{dx}$ is the force gradient of the Casimir interaction at separation *x*. By measuring the frequency shift of the cantilever for different external voltage *V*_ext_, we can calculate the real separation between two surfaces. Our measurements show that the contribution from the rms voltage fluctuations is negligible compared to the Casimir force. After canceling the contribution from electrostatic force, we can get the Casimir force gradient. The Casimir force gradient can be integrated over separation to obtain the Casimir force.

### Casimir force coupling and energy transfer

Under a slow modulation on cantilever 2, the separation between each two cantilevers is time-dependent such that:

5 $${d}_{1}(t)=	{d}_{10}-{\delta }_{{{{{{\rm{d1}}}}}}}\cos ({\omega }_{{{{{{\rm{mod1}}}}}}}t)-{\delta }_{{{{{{\rm{d2}}}}}}}\cos ({\omega }_{{{{{{\rm{mod2}}}}}}}t)\\ 	+{x}_{1}(t)-{x}_{2}(t),\\ {d}_{2}(t)=	{d}_{20}+{\delta }_{{{{{{\rm{d1}}}}}}}\cos ({\omega }_{{{{{{\rm{mod1}}}}}}}t)+{\delta }_{{{{{{\rm{d2}}}}}}}\cos ({\omega }_{{{{{{\rm{mod2}}}}}}}t)\\ 	+{x}_{2}(t)-{x}_{3}(t).$$

Here *d*_10,20_ is the equilibrium separation when there is no modulation applied, *δ*_d1,d2_ is the modulation amplitude, and *ω*_mod1,2_ are two modulation frequencies. *x*_1_(*t*), *x*_2_(*t*) and *x*_3_(*t*) describe vibrations of three cantilevers near their equilibrium positions. The motions of the cantilevers follow equations:

6 $${m}_{1}\ddot{{x}_{1}}+{m}_{1}{\gamma }_{1}\dot{{x}_{1}}+{m}_{1}{\omega }_{1}^{2}{x}_{1}=	 {F}_{C}({d}_{1}(t))\\ {m}_{2}\ddot{{x}_{2}}+{m}_{2}{\gamma }_{2}\dot{{x}_{2}}+{m}_{2}{\omega }_{2}^{2}{x}_{2}=	 -{F}_{C}({d}_{1}(t))+{F}_{C}({d}_{2}(t))\\ {m}_{3}\ddot{{x}_{3}}+{m}_{3}{\gamma }_{3}\dot{{x}_{3}}+{m}_{3}{\omega }_{3}^{2}{x}_{3}=	 -{F}_{C}({d}_{2}(t))$$

Here we generalize the displacements *x*_1,2,3_(*t*) to complex values *z*_1,2,3_(*t*) such that *x*_1,2,3_(*t*) = *Re*[*z*_1,2,3_(*t*)]. We separate the fast-rotating term and the slow-varying term for *z*_1,2,3_(*t*) such that:

7 $${z}_{1,2,3}(t)={B}_{1,2,3}(t){e}^{-i{\omega }_{1,2,3}t},$$

where *B*_1,2,3_(*t*) is the slow-varying amplitudes and we can neglect their second derivative terms ${\ddot{B}}_{1,2,3}(t)$ in the equations of motion. Under the limit of the small damping rate of three cantilevers such that *γ*_1,2,3_ ≪ *ω*_1,2,3_ and the rotating wave approximation, the equation of motion can written as:

8 $$i\left(\begin{array}{l}\dot{{B}_{1}^{\prime}}(t)\\ \dot{{B}_{2}^{\prime}}(t)\\ \dot{{B}_{3}^{\prime}}(t)\end{array}\right)=\left(\begin{array}{lll}-i\frac{{\gamma }_{1}}{2}&\frac{{{{\Lambda }}}_{1}}{4{m}_{1}{\omega }_{1}}&0\\ \frac{{{{\Lambda }}}_{1}}{4{m}_{2}{\omega }_{2}}&-i\frac{{\gamma }_{2}}{2}-{\delta }_{2}&\frac{{{{\Lambda }}}_{2}}{4{m}_{2}{\omega }_{2}}\\ 0&\frac{{{{\Lambda }}}_{2}}{4{m}_{3}{\omega }_{3}}&-i\frac{{\gamma }_{3}}{2}-{\delta }_{3}\end{array}\right)\left(\begin{array}{l}{B}_{1}^{\prime}(t)\\ {B}_{2}^{\prime}(t)\\ {B}_{3}^{\prime}(t)\end{array}\right),$$

where ${{{\Lambda }}}_{1,2}=\frac{{d}^{2}{F}_{C}}{d{x}^{2}}{|}_{{d}_{10,20}}{\delta }_{{{{{{{{\rm{d1,2}}}}}}}}}$. We have applied the transformation such that ${B}_{1}^{\prime}(t)={B}_{1}(t)$, ${B}_{2}^{\prime}(t)={B}_{2}(t){e}^{i{\delta }_{2}t}$, and ${B}_{3}^{\prime}(t)={B}_{3}(t){e}^{i{\delta }_{3}t}$, where *δ*_2_ = *ω*_1_ + *ω*_mod1_ − *ω*_2_ and *δ*_3_ = *ω*_1_ + *ω*_mod1_ − *ω*_mod2_ − *ω*_3_ are the system detunings. Under the steady condition, ${\dot{B}}_{1}$, ${\dot{B}}_{2}$, and ${\dot{B}}_{3}$ all equal to zero. The vibration amplitude of three cantilevers *A*_1,2,3_ is the absolute value of the slow-varying component so we have *A*_1,2,3_(*t*) = ∣*B*_1,2,3_(*t*)∣. In this way, the ratio of *A*_3_/*A*_1_ is:

9 $$\frac{{A}_{3}}{{A}_{1}}=\left|\frac{{B}_{3}}{{B}_{1}} \right|=\left|\frac{{{{\Lambda }}}_{1}{{{\Lambda }}}_{2}}{4{m}_{2}{m}_{3}{\omega }_{2}{\omega }_{3}{\gamma }_{2}{\gamma }_{3}+{{{\Lambda }}}_{2}^{2}} \right|.$$

The vibrations of the three cantilevers can be quantized as phonons. By introducing normalized amplitudes ${c}_{1}=\sqrt{\frac{{m}_{1}{\omega }_{1}}{\hslash }}{B}_{1}^{\prime}$, ${c}_{2}=\sqrt{\frac{{m}_{2}{\omega }_{2}}{\hslash }}{B}_{2}^{\prime}$, and ${c}_{3}=\sqrt{\frac{{m}_{3}{\omega }_{3}}{\hslash }}{B}_{3}^{\prime}$, we obtain the equation of motion for the phonon modes as:

10 $$i\left(\begin{array}{l}\dot{{c}_{1}}\\ \dot{{c}_{2}}\\ \dot{{c}_{3}}\end{array}\right)=\left(\begin{array}{lll}-i\frac{{\gamma }_{1}}{2}&\frac{{g}_{12}}{2}&0\\ \frac{{g}_{12}}{2}&-i\frac{{\gamma }_{2}}{2}-{\delta }_{2}&\frac{{g}_{23}}{2}\\ 0&\frac{{g}_{23}}{2}&-i\frac{{\gamma }_{3}}{2}-{\delta }_{3}\end{array}\right)\left(\begin{array}{l}{c}_{1}\\ {c}_{2}\\ {c}_{3}\end{array}\right),$$

where ${g}_{12}=\frac{{{{\Lambda }}}_{1}}{2\sqrt{{m}_{1}{m}_{2}{\omega }_{1}{\omega }_{2}}}=\frac{{d}^{2}{F}_{C}}{d{x}^{2}}{|}_{{d}_{10}}{\delta }_{{{{{{{{\rm{d1}}}}}}}}}\frac{1}{2\sqrt{{m}_{1}{m}_{2}{\omega }_{1}{\omega }_{2}}}$, and ${g}_{23}=\frac{{{{\Lambda }}}_{2}}{2\sqrt{{m}_{2}{m}_{3}{\omega }_{2}{\omega }_{3}}}=\frac{{d}^{2}{F}_{C}}{d{x}^{2}}{|}_{{d}_{20}}{\delta }_{{{{{{{{\rm{d2}}}}}}}}}\frac{1}{2\sqrt{{m}_{2}{m}_{3}{\omega }_{2}{\omega }_{3}}}$. Here we consider a special case that *g*_12_ = *g*_23_, *γ*_1_ = *γ*_3_, and *δ*_2,3_ = 0. The eigenvalues of the Hamiltonian are:

11 $${\lambda }_{1}=	 -i\frac{{\gamma }_{1}}{2},\\ {\lambda }_{2}=	 -i\frac{{\gamma }_{1}+{\gamma }_{2}}{4}+\frac{\sqrt{8{g}_{12}^{2}-{({\gamma }_{1}-{\gamma }_{2})}^{2}}}{4},\\ {\lambda }_{3}=	 -i\frac{{\gamma }_{1}+{\gamma }_{2}}{4}-\frac{\sqrt{8{g}_{12}^{2}-{({\gamma }_{1}-{\gamma }_{2})}^{2}}}{4}.$$

When the coupling strength is large compared to the damping difference such that $|{g}_{12}|\, > \, \frac{|{\gamma }_{1}-{\gamma }_{2}|}{2\sqrt{2}}$, we have ${{\rm{Im}}}({\lambda }_{2})=-\frac{{\gamma }_{1}+{\gamma }_{2}}{4}$ and hence the steady state requires that:

12 $${\gamma }_{1}+{\gamma }_{2} \, > \, 0.$$

When the coupling strength is small compared to damping difference such that $|{g}_{12}| < \frac{|{\gamma }_{1}-{\gamma }_{2}|}{2\sqrt{2}}$, we have ${{\rm{Im}}}({\lambda }_{2})=-\frac{{\gamma }_{1}+{\gamma }_{2}}{4}+\frac{\sqrt{{({\gamma }_{1}-{\gamma }_{2})}^{2}-8{g}_{12}^{2}}}{4}$. The steady state requires that:

13 $${\gamma }_{1}+{\gamma }_{2}-\sqrt{{({\gamma }_{1}-{\gamma }_{2})}^{2}-8{g}_{12}^{2}} \, > \, 0.$$

## Supplementary information

Supplementary Information

Description of Additional Supplementary Files

Supplementary Software

## Data Availability

All other data that support the plots within this paper and other findings of this study are available from the corresponding authors upon request. Source data are provided with this paper.

## Source data

Source Data

## Code availability

The custom codes that support the findings of this study are provided with this paper.
