# Supplementary material for: Observation and control of Casimir effects in a sphere-plate-sphere system
Source: Nat Commun. 2022 Oct 18;13:6148. doi: 10.1038/s41467-022-33915-4 (PMC9579181; doi:10.1038/s41467-022-33915-4)
Supplement: Supplementary file 1 — Supplementary Information [file 41467_2022_33915_MOESM1_ESM.pdf]

Supplementary Information for "Observation and control of Casimir effects in a sphere-plate-sphere system"

Zhujing Xu,[1] Peng Ju,[1] Xingyu Gao,[1] Kunhong Shen,[1] Zubin Jacob,[2,3] and Tongcang Li[1,2,3,4,*]

[1]*Department of Physics and Astronomy, Purdue University, West Lafayette, Indiana 47907, USA*

[2]*Elmore Family School of Electrical and Computer Engineering, Purdue University, West Lafayette, Indiana 47907, USA*

[3]*Birck Nanotechnology Center, Purdue University, West Lafayette, Indiana 47907, USA*

[4]*Purdue Quantum Science and Engineering Institute, Purdue University, West Lafayette, Indiana 47907, USA*

(Dated: October 16, 2022)

Supplementary Note 1 – Casimir interaction calculation.

In this part, we discuss how we calculate the Casimir interaction in our three-body Casimir system. We start with a two-body situation first. The Casimir force between an ideal conductive sphere and an ideal conductive plate is $F_C^0(x) = -\frac{\pi^3\hbar c}{360}\frac{R}{x^3}$, where x is the separation between two surfaces, and R is the radius of the sphere. However, the finite conductivity and dispersion of the real material needs to be considered in the calculation when we are dealing with real materials. We use the Lifshitz theory to perform the calculation [1, 2], which is briefly summarized here. The material response is characterized by its dielectric function. The plasma model for the dielectric function of gold is $\epsilon(\omega) = 1 - \frac{\omega_p^2}{\omega^2}$, where the plasma frequency ω_p is 9 eV/$\hbar$ for gold [3]. At a finite temperature, the Casimir interaction comes from both quantum and thermal fluctuations. At temperature T and separation x, the Casimir energy per unit area is given by [1, 2]

$$E(x,T) = \frac{k_B T}{2\pi}\sum_{l=0}^{\infty}{}' \int_0^{\infty} k_\perp dk_\perp \{\ln[1 - r_{TM}^2(i\xi_l, k_\perp)e^{-2xq}] + \ln[1 - r_{TE}^2(i\xi_l, k_\perp)e^{-2xq}]\}, \quad (1)$$

where $\xi_l = \frac{2\pi k_B T l}{\hbar}$ is the Matsubara frequency and $k_\perp = \sqrt{k_x^2 + k_y^2}$ is the wave vector parallel to the surface. In the summation, the $l = 0$ term will be multiplied by a factor of 1/2. The reflection coefficients of the transverse electric and magnetic mode at each Matsubara frequency and the momentum are

$$r_{TM}(i\xi_l, k_\perp) = \frac{\epsilon(i\xi_l)q(i\xi_l, k_\perp) - k(i\xi_l, k_\perp)}{\epsilon(i\xi_l)q(i\xi_l, k_\perp) + k(i\xi_l, k_\perp)}, \quad (2)$$

and

$$r_{TE}(i\xi_l, k_\perp) = \frac{q(i\xi_l, k_\perp) - k(i\xi_l, k_\perp)}{q(i\xi_l, k_\perp) + k(i\xi_l, k_\perp)}, \quad (3)$$

where $q^2(i\xi_l, k_\perp) = k_\perp^2 + \xi_l^2/c^2$ and $k^2(i\xi_l, k_\perp) = k_\perp^2 + \epsilon(i\xi_l, k_\perp)\xi_l^2/c^2$. In our experiment, the separation between two surfaces is far smaller than the dimensions of the cantilever and the sphere. Therefore, we can apply the proximity-force approximation (PFA)[4] and the Casimir force F_c between a microsphere and a cantilever is

$$F_C(x,T) = -2\pi R E(x,T). \quad (4)$$

* tcli@purdue.edu

The calculation in [2] has shown that the contribution from thermal fluctuations at room temperature is less than 4% when the separation is less than 800 nm. Thus the Casimir effect in our experiment is dominated by quantum vacuum fluctuations.

We have discussed the Casimir force between two nearby surfaces so far. Next, we will introduce the Casimir interaction in the three-body system. In our system, the thickness of the center cantilever is 1 μm, and the typical separation in our measurement is from 50 nm to 800 nm. Under such conditions, the contribution from the nonadditivity is relatively small compared to the sum of the pair potential and hence we take the additivity approximation in our calculation[5]. Under the thermal equilibrium, the force on the center cantilever can be simplified as

$$F_{2,C} = -F_C(d_1, T) + F_C(d_2, T). \tag{5}$$

where d_1 and d_2 are the separation between cantilever 1 and cantilever 2, and cantilever 2 and cantilever 3, respectively. T is the temperature of the thermal environment. Our Casimir system consists of three silicon cantilevers. Two of them are modified by attaching polystyrene spheres on them to build the sphere-plate-sphere structure. All surfaces are coated with 100-nm-thick gold films. In the calculation, we treat the interacting system as two gold spheres and a gold plate. It is indicated in [2] that the Casimir force calculation based on Supplementary Equation (1) and (4) for infinitely thick films only introduces an error less than 0.1% for 100-nm-thick thin films. Under the proximity-force approximation, the additivity approximation and the infinitely thick gold film approximation, we can calculate the Casimir force on the center cantilever based on Supplementary Equation (1), (4), and (5) without introducing much error.

To better understand the three-body Casimir system, we also performed more advanced calculation for three-body Casimir force without the additivity approximation. Here we discuss the effect of the thickness of the center plate on the nonadditivity component. The configuration is shown in the inset of Supplementary Figure 1.(a). The Casimir system consists of three gold plates. To study the Casimir force on the left gold plate(L), we treat the system as a gold plate(L) and a multi-layer structure and they are separated by a

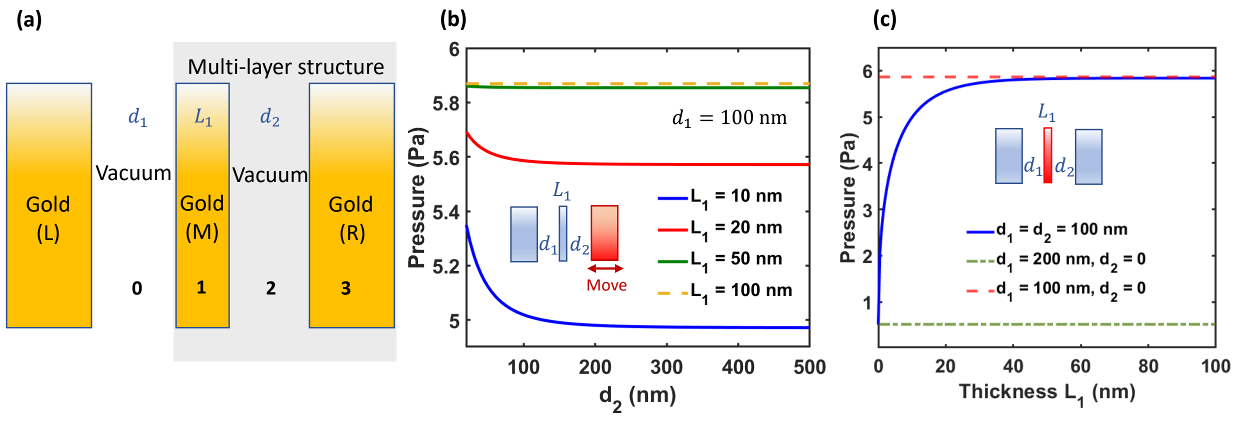

Supplementary Figure 1. Calculation of Casimir force for a multi-layer system. (a). We treat our Casimir system as a left gold plate(L) and a multi-layer system and calculate the pressure on the gold plate(L). The multi-layer structure consists of three layers which are a middle gold membrane(M), a vacuum and a right gold plate(R). The interface between the gold plate(L) and the multi-layer structure is vacuum. The left gold plate(L) and the right gold plate(R) are assumed to be infinitely thick. (b). The calculated Casimir pressure on the gold plate(L) is shown as a function of d_2 for different membrane thickness L_1. Here d_1 is set to be 100 nm. When the thickness of the gold membrane(M) is 10 nm, the Casimir pressure on the gold plate(L) changes by 7.6 % when the separation d_2 increases from 20 nm to 500 nm. If the thickness of the gold membrane(M) is 100 nm, the Casimir pressure on the gold plate(L) only changes by 9×10^{-4} % when d_2 changes from 20 nm to 500 nm. (c). The calculated Casimir pressure on the gold plate(L) is shown as a function of the thickness L_1 while the separation d_1 and d_2 are fixed. When the thickness L_1 is larger than 50 nm, the direct Casimir force interaction between the gold plate(L) and the gold plate(R) is negligible.

distance d_1 in vacuum. The multi-layer structure consists of three layers which are a thin gold membrane with a thickness of L_1, a vacuum with thickness d_2 and a infinitely thick

gold plate. The Casimir pressure on the gold plate(L) is given by[6]

$$P(x,T) = -\frac{k_B T}{\pi} \sum_{l=0}^{\infty}{}' \int_0^\infty q k_\perp dk_\perp \{ [r_{1\mathrm{p}}^{-1}(i\xi_l,k_\perp) r_{2\mathrm{p}}^{-1}(i\xi_l,k_\perp) e^{2xq} - 1]^{-1} + [r_{1\mathrm{s}}^{-1}(i\xi_l,k_\perp) r_{2\mathrm{s}}^{-1}(i\xi_l,k_\perp) e^{2xq} - 1]^{-1} \}, \tag{6}$$

Here $r_{1\mathrm{p}}$ and $r_{1\mathrm{s}}$ are the reflection coefficients of the gold plate(L) for the p and s polarization and they are written as

$$r_{1\mathrm{p}}(i\xi_l,k_\perp) = \frac{\epsilon(i\xi_l) q(i\xi_l,k_\perp) - k(i\xi_l,k_\perp)}{\epsilon(i\xi_l) q(i\xi_l,k_\perp) + k(i\xi_l,k_\perp)}, \tag{7}$$

and

$$r_{1\mathrm{s}}(i\xi_l,k_\perp) = \frac{q(i\xi_l,k_\perp) - k(i\xi_l,k_\perp)}{q(i\xi_l,k_\perp) + k(i\xi_l,k_\perp)}. \tag{8}$$

$r_{2\mathrm{p}}$ and $r_{2\mathrm{s}}$ are the reflection coefficients of the multi-layer structure for the p and s polarization and we introduce the transfer matrix method to describe them. Here the transfer matrix for the $p(s)$ polarization is written as [6, 7]

$$M^{p(s)} = D_{0\to1}^{p(s)} P_1(L_1) D_{1\to2}^{p(s)} P_2(d_2) D_{2\to3}^{p(s)}, \tag{9}$$

where each layer is labeled as $0, 1, 2, 3$ as shown in Supplementary Figure 4.(a). $D_{j\to j+1}^{p(s)}$ is the transmission matrix between layer j and $j+1$ for the $p(s)$ polarization and is given as

$$D_{j\to j+1}^{p(s)} = \frac{1}{2} \begin{bmatrix} 1+\eta_{j,j+1}^{p(s)} & 1-\eta_{j,j+1}^{p(s)} \\ 1-\eta_{j,j+1}^{p(s)} & 1+\eta_{j,j+1}^{p(s)} \end{bmatrix}, \tag{10}$$

where $\eta_{j,j+1}^{p(s)}$ is written as

$$\eta_{j,j+1}^{p} = \frac{\epsilon_j(i\xi) K_{j+1}}{\epsilon_{j+1}(i\xi) K_j}, \quad \eta_{j,j+1}^{s} = \frac{K_{j+1}}{K_j}. \tag{11}$$

Here $K_j = \sqrt{k_\perp^2 + \epsilon_j(i\xi)\xi^2/c^2}$. $P_1(L_1)$ and $P_2(d_2)$ are the propagation matrix for layer 1 and layer 2 and they are given as

$$P_1(L_1) = \begin{bmatrix} e^{K_1 L_1} & 0 \\ 0 & e^{-K_1 L_1} \end{bmatrix}, \tag{12}$$

and

$$P_2(d_2) = \begin{bmatrix} e^{K_2 d_2} & 0 \\ 0 & e^{-K_2 d_2} \end{bmatrix}. \tag{13}$$

We can then get the reflection coefficients of the multi-layer structure as

$$r_{2p(s)} = M_{21}^{p(s)} / M_{11}^{p(s)}, \tag{14}$$

where $M_{11}^{p(s)}$ and $M_{21}^{p(s)}$ are the components of the transfer matrix $M^{p(s)}$ based on Supplementary Equation (10).

The calculated Casimir pressure on the gold plate(L) is shown in Supplementary Figure 1.(b). When the thickness of the middle gold membrane(M) is 100 nm, the Casimir pressure on the gold plate(L) only changes by 9×10^{-4} % when d_2 changes from 20 nm to 500 nm. Here we consider a case that $d_1 = 100$ nm. In our experiment, the thickness of the middle gold membrane (center gold-coated silicon cantilever) is about 1μm, and hence the direct Casimir force interaction between the left cantilever and the right cantilever is negligible in our system.

Supplementary Note 2 – Experimental set-up and force measurement.

In our experiment, we built a three-cantilever Casimir system, as shown in Supplementary Figure 2.(a). We modified all three cantilevers. We put a 70-μm-diameter polystyrene microsphere on top of the left and the right cantilevers for creating a sphere-plate-sphere structure. All surfaces are coated with 100-nm gold layers. Three independent fiber interferometers are implemented to monitor the motion of each cantilever. We can use the piezo chips to drive three cantilevers independently and change the separations between each two surfaces. The inset of Supplementary Figure 2.(a) shows the optical image of the Casimir system. We also show the scanning electron microscope (SEM) image of the sphere-cantilever and the bare cantilever in Supplementary Figure 2.(b) and (c). We coat the cantilevers with gold films by an E-beam evaporator. The recorded power spectrum densities (PSD) of three cantilevers are shown in Supplementary Figure 3.

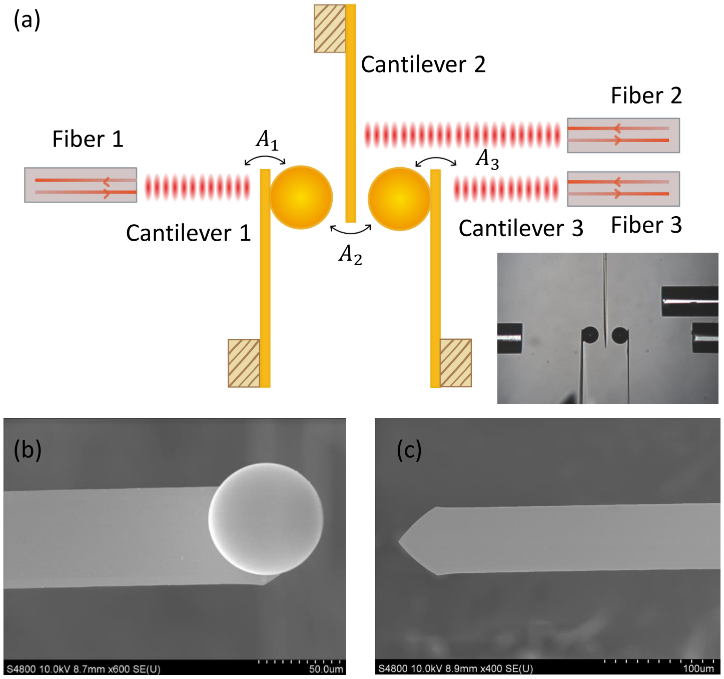

Supplementary Figure 2. Experimental setup of the Casimir system. (a). Schematics of the three-cantilever Casimir setup. The motion of three cantilevers are detected by three independent fiber interferometers. The inset shows an optical image of the system. (b) and (c). Scanning electron microscope (SEM) image of a modified cantilever-sphere system and a bare cantilever. Both surfaces are coated with 100-nm gold layers.

In the experiment, we apply dynamic force measurement scheme and phase-lock loop (PLL) to track the resonant frequency in the presence of the Casimir interaction. Then we can get the force gradient as $\frac{dF}{dx} = -2k\frac{\delta\omega}{\omega}$, where k is the spring constant of the cantilever, $\delta\omega$ is the frequency shift in the presence of the interaction and ω is the natural resonant frequency. The separation between each two surfaces is calibrated by the electrostatic force. The frequency shift due to the electrostatic force and the Casimir force is $\Delta\omega = -\frac{\omega}{2k}\frac{\pi\epsilon_0 R}{x^2}[(V_{\text{ext}}-V_c)^2+V_{\text{rms}}^2]-\frac{\omega}{2k}\frac{dF_C}{dx}$, where V_{ext} is the external voltage applied on the surface, V_c is the patch potential, V_{rms} is the rms voltage fluctuations. $\frac{dF_C}{dx}$ is the force gradient due to the Casimir interaction at separation x. Our measurements show that the contribution from rms voltage fluctuations

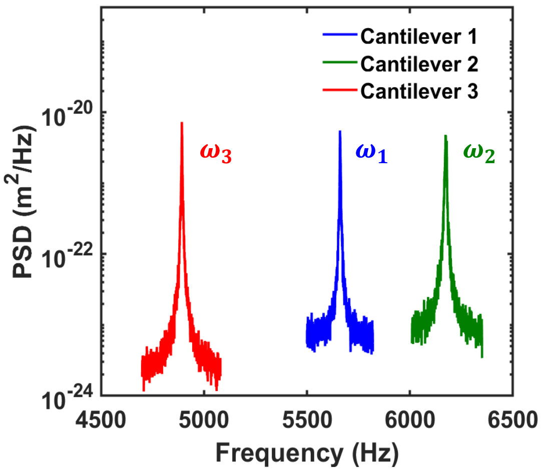

Supplementary Figure 3. Power spectrum density (PSD) of three cantilevers in our system.

is negligible compared to the Casimir interaction. After canceling the contribution from the electrostatic force, we can get the Casimir force gradient $\frac{dF_c}{dx}$ at each separation.

Here we introduce how we get the force gradient as shown in FIG.1 in the main text. Supplementary Figure 4.(a) shows the frequency shift of cantilever 2 as a function of the externally applied voltage between two surfaces when cantilever 3 is placed nearby and cantilever 1 is far away. By fitting the data with a parabolic function, we can get a calibrated separation of $d_2 = 310$ nm for Fig.1.(d) in the main text. By fixing the separation d_2 while moving cantilever 1 to a closer position, we can measure the frequency shift as a function of d_1, as shown in Supplementary Figure 4.(b). The red diamonds are the total frequency shift due to the Casimir interaction, including the contribution from both cantilever 1 and cantilever 3. The blue circles are the contribution only from cantilever 1. The measured frequency shift gives the information of the force gradient by $\frac{dF}{dx} = -2k\frac{\delta\omega}{\omega}$ as shown in Fig.1.(d) in the main text. We also show a measured patch potential between cantilever 1 and cantilever 2 at each separation d_1. The patch potential is around -5 mV. The frequency shift from patch potential is far smaller than the contribution from Casimir interaction. Similarly, the calibrated separation d_1, the measured frequency shift and the measured patch potential for

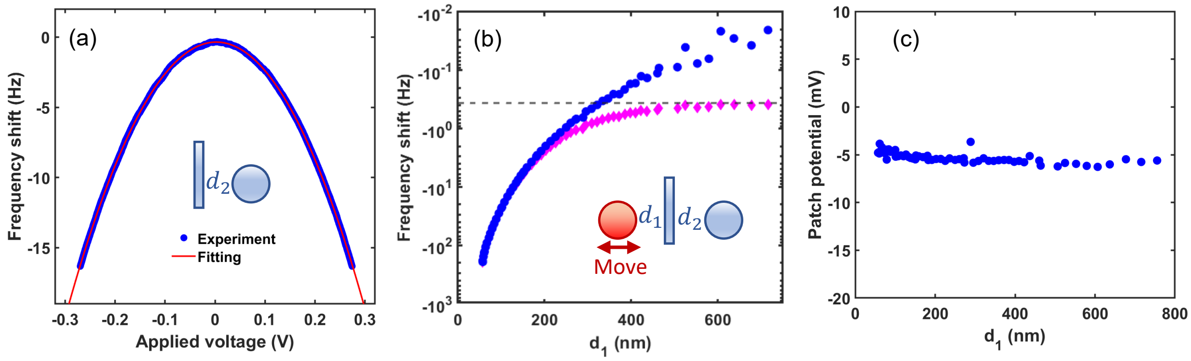

Supplementary Figure 4. Measurement of the frequency shift and the patch potential by electrostatic calibration when d_1 is changing. (a). When cantilever 1 is far away, the measured frequency shift of cantilever 2 in the presence of Casimir force and electrostatic force between cantilever 2 and 3 is shown as a function of the applied voltage. The parabolic fitting gives the calibrated separation $d_2 = 310$ nm. (b). By extracting the data when the patch potential is compensated, the frequency shift from Casimir interaction is shown as a function of the separation d_1. (c). Measured patch potential between cantilever 1 and cantilever 2 is shown as a function of separation d_1.

Fig.1.(e) in the main text are shown in Supplementary Figure 5.

Supplementary Note 3 – External gain to the system

Now we introduce how we add external gain to the system to realize the amplification of energy transfer by quantum vacuum fluctuations. In the experiment, we add extra gain to cantilever 2 by feedback control as shown in Supplementary Figure 6.(a). Under the feedback control, the damping rate of cantilever 2 becomes $\gamma_2 = \gamma_{20} - G$, where γ_{20} is the natural damping rate of cantilever 2 and G is the gain coefficient.

When $G < \gamma_{20}$, the damping rate of cantilever 2 is still positive. We use the ringdown scheme to calibrate the damping rate under such conditions as shown in Supplementary Figure 6.(b). The cantilever 2 is first driven resonantly to a large amplitude. At Time = 0, we turn off the driving voltage and record the displacement x_2 as a function of time. By extracting the

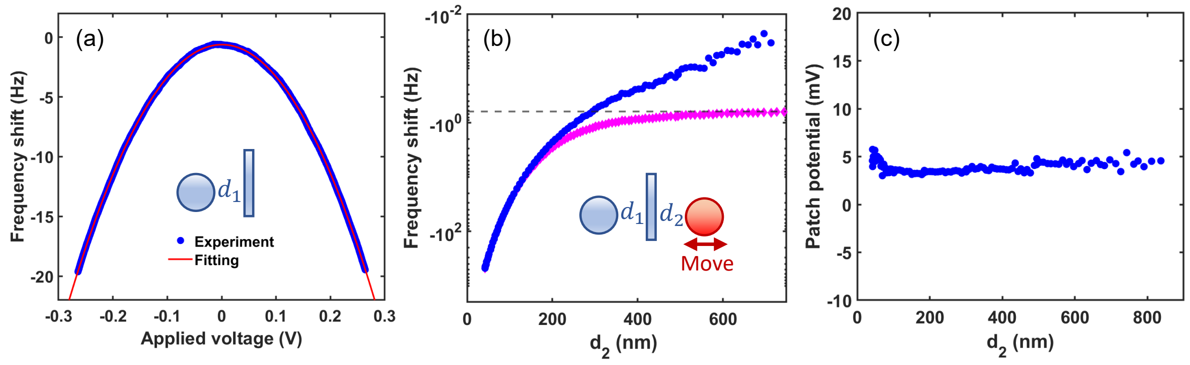

Supplementary Figure 5. Measurement of the frequency shift and the patch potential by electrostatic calibration when d_2 is changing. (a). When cantilever 3 is far away, the measured frequency shift of cantilever 2 in the presence of Casimir force and electrostatic force between cantilever 1 and 2 is shown as a function of the applied voltage. The parabolic fitting gives the calibrated separation $d_1 = 276$ nm. (b). By extracting the data when the patch potential is compensated, the frequency shift from Casimir interaction is shown as a function of the separation d_2. (c). Measured patch potential between cantilever 2 and cantilever 3 is shown as a function of separation d_2.

envelope of the oscillating dispalcement and fit it with a function $x_2 = A \times \exp(-\frac{\gamma_2}{2}t)$, we can calibrate the real damping rate γ_2 under a gain as shown in Supplementary Figure 6.(c). When $G > \gamma_{20}$, the damping rate of cantilever 2 is negative. When we turn on the gain at Time $= 0$, the motion of cantilever 2 will be amplified as a function of time as shown in Supplementary Figure 6.(d). We fit the envelope with the same function that $A \times \exp(-\frac{\gamma_2}{2}t)$ to extract the damping rate γ_2 here.

In the experiment, we add gain by a derivative function in the PID control. The calibrated γ_2 at each derivative control parameter $K_c * T_d/T_s$ is shown in Supplementary Figure 6.(f), where K_c is the proportional gain, T_d is the derivative time and T_s is the PID loop time. In our system, the PID loop rate is 50000 Hz. We can control γ_2 from $2\pi \times 6$ Hz to about γ_2 from -$2\pi \times 6$ Hz. A larger gain will lead to instability of the three-cantilever system. The requirement of the steady condition will be discussed in the next session.

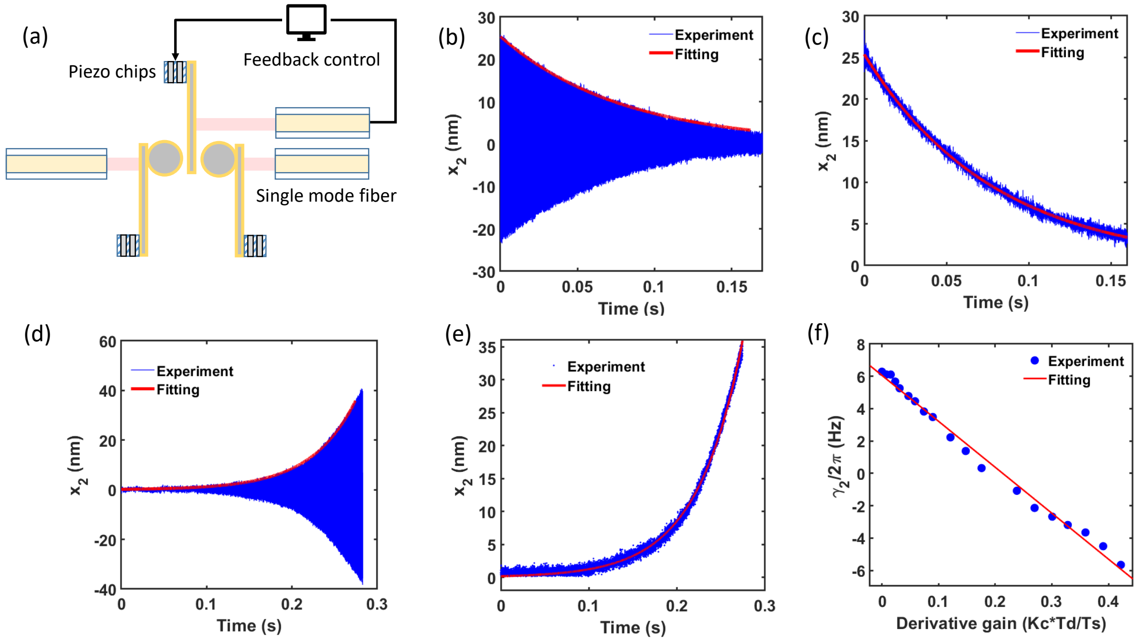

Supplementary Figure 6. Extra gain is applied to cantilever 2 by a PID feedback control loop. (a). Schematic of the three-cantilever and feedback control system. The motion of cantilever 2 is recorded from the fiber interferometer and is sent to the computer. The computer does the derivative of the signal and drive the piezo chips accordingly. (b) and (c). We apply a gain to the system such that $\gamma_2 = \gamma_{20} - G > 0$. (b). We use ringdown scheme for damping rate γ_2 calibration. We first drive cantilever 2 resonantly to a large amplitude. We turn off the driving voltage and turn on the feedback control(gain) at Time $= 0s$. (c). We extract the upper envelope of (b) and fit the amplitude by a function $x_2 = A \times \exp(-\frac{\gamma_2}{2}t)$ and get the value γ_2. (d) and (e). We apply a gain to the system such that $\gamma_2 = \gamma_{20} - G < 0$. We turn on the feedback control(gain) at Time $= 0s$ and fit the envelope with the same function $x_2 = A \times \exp(-\frac{\gamma_2}{2}t)$ to extract the negative damping rate γ_2. (f). The calibrated damping rate γ_2 is shown at each derivative gain value in the PID control.

Supplementary Note 4 – Casimir force coupling and energy transfer

In this session, we will discuss the Casimir coupling in our three-body system. Under a slow modulation on cantilever 2, the separation between each two cantilevers are time-dependent such that

$$
\begin{aligned}
d_1(t) &= d_{10} - \delta_{\rm d1}\cos(\omega_{\rm mod1}t) - \delta_{\rm d2}\cos(\omega_{\rm mod2}t) + x_1(t) - x_2(t), \\
d_2(t) &= d_{20} + \delta_{\rm d1}\cos(\omega_{\rm mod1}t) + \delta_{\rm d2}\cos(\omega_{\rm mod2}t) + x_2(t) - x_3(t).
\end{aligned} \tag{15}
$$

Here $d_{10,20}$ is the equilibrium separation when there is no modulation applied, $\delta_{\rm d1,d2}$ is the modulation amplitude, and $\omega_{\rm mod1,2}$ are two modulation frequencies. $x_1(t)$, $x_2(t)$ and $x_3(t)$ describe vibrations of three cantilevers near their equilibrium positions. The motions of the cantilevers follow equations

$$
\begin{aligned}
m_1\ddot{x_1} + m_1\gamma_1\dot{x_1} + m_1\omega_1^2 x_1 &= F_C(d_1(t)), \\
m_2\ddot{x_2} + m_2\gamma_2\dot{x_2} + m_2\omega_2^2 x_2 &= -F_C(d_1(t)) + F_C(d_2(t)), \\
m_3\ddot{x_3} + m_3\gamma_3\dot{x_3} + m_3\omega_3^2 x_3 &= -F_C(d_2(t)).
\end{aligned} \tag{16}
$$

When the separation between each surfaces is far larger than the modulation amplitude and the oscillation amplitude of three cantilevers such that $d_{10,20} \gg \delta_d$, x_1, x_2, the Casimir force term $F_C(d_{1,2})$ can be expanded to the second order with respect to the term $-\delta_{\rm d1}\cos(\omega_{\rm mod1}t) - \delta_{\rm d2}\cos(\omega_{\rm mod2}t) + x_1(t) - x_2(t)$ and $\delta_{\rm d1}\cos(\omega_{\rm mod1}t) + \delta_{\rm d2}\cos(\omega_{\rm mod2}t) + x_2(t) - x_3(t)$. In our experiment, we utilize parametric modulation to couple each two cantilevers and the direct coupling is neglected since the coupling strength is far smaller than the frequency differences between each two cantilevers in our experiment (about 470 Hz and 1230 Hz). The zero-order and first-order terms of $F_C(d_{1,2})$ shift the frequency of the cantilevers but does not contribute to the energy transfer since they are off-resonant. The contribution comes from the second derivative of the Casimir force $F_C(d_{1,2})$. Under the limit of small modulation amplitudes and small oscillation amplitudes of three cantilevers,

the equations can be written as

$$\begin{aligned}
\ddot{x_1} + \gamma_1 \dot{x_1} + \omega_1^2 x_1 &= \frac{\Lambda_1}{m_1} \cos(\omega_{\text{mod1}} t)(x_1 - x_2). \\
\ddot{x_2} + \gamma_2 \dot{x_2} + \omega_2^2 x_2 &= \frac{\Lambda_1}{m_2} \cos(\omega_{\text{mod1}} t)(x_2 - x_1) \\
&+ \frac{\Lambda_2}{m_2} \cos(\omega_{\text{mod2}} t)(x_2 - x_3). \\
\ddot{x_3} + \gamma_3 \dot{x_3} + \omega_3^2 x_3 &= \frac{\Lambda_2}{m_3} \cos(\omega_{\text{mod2}} t)(x_3 - x_2).
\end{aligned} \tag{17}$$

Here we have $\Lambda_{1,2} = \frac{d^2 F_C}{dx^2}|_{d_{10,20}} \delta_{\text{d}1,2}$.

We now generalize the displacements $x_{1,2,3}(t)$ to complex values $z_{1,2,3}(t)$ such that $x_{1,2,3}(t) = Re[z_{1,2,3}(t)]$. We separate the fast-rotating term and the slow-varying term for $z_{1,2,3}(t)$ to solve Supplementary Equation (17) such that

$$z_{1,2,3}(t) = B_{1,2,3}(t) e^{-i\omega_{1,2,3} t}, \tag{18}$$

where $B_{1,2,3}(t)$ is the slow-varying oscillating component for three cantilevers and hence we can neglect their second derivative terms $\ddot{B}_{1,2,3}(t)$. Under the limit of the small damping rate of three cantilevers such that $\gamma_{1,2,3} \ll \omega_{1,2,3}$, the equations of motion can be written as

$$\begin{aligned}
&-i\omega_1 \gamma_1 B_1(t) e^{-i\omega_1 t} - 2i\omega_1 \dot{B}_1(t) e^{-i\omega_1 t} \\
&= \frac{\Lambda_1}{2m_1} (B_1(t) e^{-i(\omega_1 + \omega_{\text{mod1}})t} - B_2(t) e^{-i(\omega_2 - \omega_{\text{mod1}})t}), \\
&-i\omega_2 \gamma_2 B_2(t) e^{-i\omega_2 t} - 2i\omega_2 \dot{B}_2(t) e^{-i\omega_2 t} \\
&= \frac{\Lambda_1}{2m_2} (B_2(t) e^{-i(\omega_2 - \omega_{\text{mod1}})t} - B_1(t) e^{-i(\omega_1 + \omega_{\text{mod1}})t}) \\
&+ \frac{\Lambda_2}{2m_2} (B_2(t) e^{-i(\omega_2 - \omega_{\text{mod2}})t} - B_3(t) e^{-i(\omega_3 + \omega_{\text{mod2}})t}), \\
&-i\omega_3 \gamma_3 B_3(t) e^{-i\omega_3 t} - 2i\omega_3 \dot{B}_3(t) e^{-i\omega_3 t} \\
&= \frac{\Lambda_2}{2m_3} (B_3(t) e^{-i(\omega_3 + \omega_{\text{mod2}})t} - B_2(t) e^{-i(\omega_2 - \omega_{\text{mod2}})t}),
\end{aligned} \tag{19}$$

where the rotating-frame approximation is taken and the fast-rotating terms are neglected.

Now we apply the transformation such that $B'_1(t) = B_1(t)$, $B'_2(t) = B_2(t) e^{i\delta_2 t}$, and $B'_3(t) = B_3(t) e^{i\delta_3 t}$, where $\delta_2 = \omega_1 + \omega_{\text{mod1}} - \omega_2$ and $\delta_3 = \omega_1 + \omega_{\text{mod1}} - \omega_{\text{mod2}} - \omega_3$. Then, the equation

of motion becomes

$$i\begin{pmatrix}\dot{B}_1'(t)\\ \dot{B}_2'(t)\\ \dot{B}_3'(t)\end{pmatrix}=\begin{pmatrix}-i\frac{\gamma_1}{2} & \frac{\Lambda_1}{4m_1\omega_1} & 0\\ \frac{\Lambda_1}{4m_2\omega_2} & -i\frac{\gamma_2}{2}-\delta_2 & \frac{\Lambda_2}{4m_2\omega_2}\\ 0 & \frac{\Lambda_2}{4m_3\omega_3} & -i\frac{\gamma_3}{2}-\delta_3\end{pmatrix}\begin{pmatrix}B_1'(t)\\ B_2'(t)\\ B_3'(t)\end{pmatrix}, \tag{20}$$

where we have neglected the fast-rotating terms. Under the steady condition, $\dot{B}_1$, $\dot{B}_2$, and $\dot{B}_3$ all equal to zero. The vibration amplitude of three cantilevers is the absolute value of the slow-varying component such that $A_{1,2,3}(t) = |B_{1,2,3}(t)|$. When cantilever 1 is driven resonantly at ω_1 and the parametric modulation is on resonance such that $\delta_2 = \delta_3 = 0$, the ratio of A_3/A_1 is

$$\frac{A_3}{A_1} = |\frac{B_3}{B_1}| = |\frac{\Lambda_1\Lambda_2}{4m_2m_3\omega_2\omega_3\gamma_2\gamma_3+\Lambda_2^2}|. \tag{21}$$

In the strong coupling regime that $|g_{23}| = |\frac{\Lambda_2}{2\sqrt{m_2m_3\omega_2\omega_3}}| \gg |\gamma_{2,3}|$, the transduction amplitude is approximated to be

$$\frac{A_3}{A_1} = |\frac{\Lambda_1}{\Lambda_2}|. \tag{22}$$

Under the weak coupling limit that $|g_{23}| = |\frac{\Lambda_2}{2\sqrt{m_2m_3\omega_2\omega_3}}| \ll |\gamma_{2,3}|$, the transduction amplitude is approximated to be

$$\frac{A_3}{A_1} = |\frac{\Lambda_1\Lambda_2}{4m_2m_3\omega_2\omega_3\gamma_2\gamma_3}|. \tag{23}$$

We can quantize the oscillations of the three cantilevers as phonons. By introducing normalized amplitudes $c_1 = \sqrt{\frac{m_1\omega_1}{\hbar}}B_1'$, $c_2 = \sqrt{\frac{m_2\omega_2}{\hbar}}B_2'$, and $c_3 = \sqrt{\frac{m_3\omega_3}{\hbar}}B_3'$, we obtain the equation of motion for the phonon modes as

$$i\begin{pmatrix}\dot{c_1}\\ \dot{c_2}\\ \dot{c_3}\end{pmatrix}=\begin{pmatrix}-i\frac{\gamma_1}{2} & \frac{g_{12}}{2} & 0\\ \frac{g_{12}}{2} & -i\frac{\gamma_2}{2}-\delta_2 & \frac{g_{23}}{2}\\ 0 & \frac{g_{23}}{2} & -i\frac{\gamma_3}{2}-\delta_3\end{pmatrix}\begin{pmatrix}c_1\\ c_2\\ c_3\end{pmatrix}, \tag{24}$$

where $g_{12} = \frac{\Lambda_1}{2\sqrt{m_1m_2\omega_1\omega_2}} = \frac{d^2F_C}{dx^2}|_{d_{10}}\delta_{d1}\frac{1}{2\sqrt{m_1m_2\omega_1\omega_2}}$, and $g_{23} = \frac{\Lambda_2}{2\sqrt{m_2m_3\omega_2\omega_3}} = \frac{d^2F_C}{dx^2}|_{d_{20}}\delta_{d2}\frac{1}{2\sqrt{m_2m_3\omega_2\omega_3}}$. Therefore, the effective Hamiltonian of the system can be rewritten as

$$H=\begin{pmatrix}-i\frac{\gamma_1}{2} & \frac{g_{12}}{2} & 0\\ \frac{g_{12}}{2} & -i\frac{\gamma_2}{2}-\delta_2 & \frac{g_{23}}{2}\\ 0 & \frac{g_{23}}{2} & -i\frac{\gamma_3}{2}-\delta_3\end{pmatrix}. \tag{25}$$

Now we consider a special case that $g_{12} = g_{23}$, $\gamma_1 = \gamma_3$, and $\delta_{2,3} = 0$. The eigenvalues of the Hamiltonian are

$$\begin{aligned}
\lambda_1 &= -i\frac{\gamma_1}{2}, \\
\lambda_2 &= -i\frac{\gamma_1 + \gamma_2}{4} + \frac{\sqrt{8g_{12}^2 - (\gamma_1 - \gamma_2)^2}}{4}, \\
\lambda_3 &= -i\frac{\gamma_1 + \gamma_2}{4} - \frac{\sqrt{8g_{12}^2 - (\gamma_1 - \gamma_2)^2}}{4}.
\end{aligned} \tag{26}$$

When the coupling strength is large compared to the damping difference such that $|g_{12}| > \frac{|\gamma_1 - \gamma_2|}{2\sqrt{2}}$, we have $Im(\lambda_2) = -\frac{\gamma_1+\gamma_2}{4}$ and hence the steady state requires that

$$\gamma_1 + \gamma_2 \geq 0. \tag{27}$$

When the coupling strength is small compared to damping difference such that $|g_{12}| < \frac{|\gamma_1 - \gamma_2|}{2\sqrt{2}}$, we have $Im(\lambda_2) = -\frac{\gamma_1+\gamma_2}{4} + \frac{\sqrt{(\gamma_1-\gamma_2)^2 - 8g_{12}^2}}{4}$. The steady state requires that

$$\gamma_1 + \gamma_2 - \sqrt{(\gamma_1 - \gamma_2)^2 - 8g_{12}^2} \geq 0. \tag{28}$$

In a special case that $\gamma_1 = \gamma_2 = \gamma_3 = 0$, $g_{12} = g_{23} = g$, and $\delta_{2,3} = 0$, the eigenvalues of the Hamiltonian are $\lambda_1 = 0, \lambda_2 = g/\sqrt{2}, \lambda_3 = -g/\sqrt{2}$. The corresponding eigenvectors are:

$$|e_1\rangle = \frac{1}{\sqrt{2}}\begin{pmatrix} 1 \\ 0 \\ -1 \end{pmatrix}, \; |e_2\rangle = \frac{1}{2}\begin{pmatrix} 1 \\ \sqrt{2} \\ 1 \end{pmatrix}, \; |e_3\rangle = \frac{1}{2}\begin{pmatrix} 1 \\ -\sqrt{2} \\ 1 \end{pmatrix}. \tag{29}$$

Interestingly, $|e_1\rangle$ corresponds to the case when cantilever 1 and cantilever 3 oscillate with the same amplitude, but cantilever 2 does not oscillate at all. Thus only two modes will show up in the motion of cantilever 2 under these special conditions, as expected in FIG. 3.(c) and (d) in the main text.

Supplementary References

---

[1] E. M. Lifshitz, The theory of molecular attractive forces between solids, Sov. Phys. JETP **2**, 73 (1956).

[2] Z. Xu, X. Gao, J. Bang, Z. Jacob, and T. Li, Non-reciprocal energy transfer through the casimir effect, Nature Nanotechnology **17**, 148 (2022).

[3] E. D. Palik, *Handbook of Optical Constants of Solids* (Academic Press, Boston, 1998) p. iii.

[4] J. Błocki, J. Randrup, W. J. Światecki, and C. F. Tsang, Proximity forces, Annals of Physics **105**, 427 (1977).

[5] R. Messina and M. Antezza, Three-body radiative heat transfer and casimir-lifshitz force out of thermal equilibrium for arbitrary bodies, Phys. Rev. A **89**, 052104 (2014).

[6] L. Ge, X. Shi, Z. Xu, and K. Gong, Tunable casimir equilibria with phase change materials: From quantum trapping to its release, Phys. Rev. B **101**, 104107 (2020).

[7] T. Zhan, X. Shi, Y. Dai, X. Liu, and J. Zi, Transfer matrix method for optics in graphene layers, Journal of Physics: Condensed Matter **25**, 215301 (2013).
