# Supplementary material for: Observation and control of Casimir effects in a sphere-plate-sphere system
Source: Nat Commun. 2022 Oct 18;13:6148. doi: 10.1038/s41467-022-33915-4 (PMC9579181; doi:10.1038/s41467-022-33915-4)
Supplement: Supplementary file 2 — Description of Additional Supplementary Files [file 41467_2022_33915_MOESM2_ESM.pdf]

Supplementary Software: Custom Matlab codes to calculate the Casimir interaction in the sphereplate-sphere system.
